# New data on recently described rodent species Paulina's Limestone Rat *Saxatilomys paulinae* Musser, Smith, Robinson & Lunde, 2005 (Mammalia: Rodentia)

**DOI:** 10.3897/BDJ.3.e4961

**Published:** 2015-05-19

**Authors:** Nghia Xuan Nguyen, Dang Xuan Nguyen, Tuong Xuan Ngo, Duy Dinh Nguyen

**Affiliations:** ‡ Institute of Ecology and Biological Resources, VAST, Hanoi, Vietnam

**Keywords:** Rodent, morphology, Quang Binh, Khammouane, karst limestone forests, Central Indochina Limestone

## Abstract

**Background:**

Paulina's Limestone Rat *Saxatilomys paulinae* Musser et al., 2005 was first discovered by Musser et al. (2005) based on specimens from the Khammouane Limestone National Biodiversity Conservation Area (NBCA) in Khammouane Province in central Lao People's Democratic Republic (PDR). This tower karst landscape is part of the Central Indochina Limestone massif, which extends eastward into north-central Vietnam in Quang Binh and Quang Tri Provinces.

**New information:**

In April 2014, we conducted a rodent survey and collected four (4) whole specimens of *Saxatilomys paulinae* in Quang Binh province. This is the first record of *Saxatilomys paulinae* in Vietnam. External and craniodental characteristics of all specimens clearly exhibit the characters of *Saxatilomys paulinae* as described in Musser et al. (2005)​. The rats are of medium size (HB: 160.3 ± 2.03 mm, T: 192.3 ± 6.69 mm) with some specific morpological characteristics. The external and craniodental measurement of the specimens from Vietnam tend to be larger than those of specimens from Lao. However, this needs to be verified by more studies in future. The habitat of *Saxatilomys paulinae* in Vietnam is characterized by complicated terrain comprising low karst towers (around 400 m) with steep slopes covered under limestone humid evergreen forest. The forest has been affected by selected timber logging in the past, but still has a complex 4-layer structure. The population of *Saxatilomys paulinae* in Vietnam is threatened by rodent trapping/snaring and habitat disturbance. More status surveys should be conducted to assess the species distributional range and its population status for undertaking relevant conservation measures.

## Introduction

The Central Indochina Limestone is the most extensive tracts of limestone karst habitat in Indochina (Sterling et al. 2006) which straddles the border between Vietnam (Quang Binh and Quang Tri Provinces) and Lao People's Democratic Republic (Khammouane Province). This landscape harbors an endemicity in birds and mammals which would appear to exceed that of other limestone areas of Southeast Asia (Baltzer et al. 2001, Tordoff et al. 2003). Recent studies have discovered two new rodent species: Laotian Rock Rat *Laonastes aenigmamus* (Jenkins et al. 2005) and Paulina's Limestone Rat *Saxatilomys paulinae* (Musser et al. 2005) in Khammouane Limestone (NBCA) in Khammouane Province. *Laonastes aenigmamus* is listed in IUCN Red List as Endangered while *Saxatilomys paulinae* as Data Deficient as this is a very recently described species and the limits of its disrtibution are not yet well known. There is a need for further survey work to determine whether it might occur more widely than current records suggest, and its population status (Lunde and Musser 2008). Providing that comparable environments are extant in Quang Binh province (Vietnam), we expected that these two species would inhabit also Quang Binh limestone. Thus, from 2011 to 2014, we conducted extensive rodent surveys and collected specimens of both, *Laonastes aenigmamus* and *Saxatilomys paulinae*, in the area. These are the first records of *Laonastes aenigmamus* and *Saxatilomys paulinae* in Vietnam. We have published the data on *Laonastes aenigmamus* in Dang et al. (2012) and Nguyen et al. (2014). This paper provides new data on morphology and habitat characteristics of *Saxatilomys paulinae* in Vietnam.

## Materials and methods

Quang Binh limestone habitat is characterized by very specific natural conditions of precipitous karst ridges, which rise to elevations of around 400 m. Scattered among this ridges are narrow valleys and pockets of igneous rock formations. The limestone karst is almost entirely forested, apart from steep cliff faces. The forest clearance occurs only in flat valleys within the limestone massif and in lowland area bordering it. The most widespread forest type in the landscape is limestone evergreen forest, but there are also significant areas of lowland evergreen forest distributed on non-calcareous substrate in valleys among the limestone karsts (Le et al. 2012). The climate in the area is tropical, hot and humid. The annual mean temperature ranges between 23 °C and 25 °C, with summer maximum of 41 °C and winter minimum of 6 °C. The high annual rainfall averages 2,000 - 2,500 mm, 88% falling between July and December, though there is rain in every month. The mean annual relative humidity is 84% (Le et al. 2012). Because of limestone topography, drainage is complex and there area few permanent water sources.

We used Sherman live traps and Tomahawk live traps to sample the rodent specimens. At each survey location, trap-transects were established within various habitat types and contain 30-50 traps each depending on habitat type. Traps were set at each transect for 4–6 days and checked on subsequent mornings (for specimens and for bait replacement). Baits were made of fresh manioc or sweet potato. All captured animals were anesthetized and measured for external measurements following Lunde and Le (2001): head-body length (HB), tail length (T), hind-foot length (HF), and ear length (E) (in mm), and weight (W) (in g). Captured animals were identified, sexed and the time of capture recorded. After examination, captured rodents were released immediately at capture point.

Those individuals that could not be identified to the species level in the field (this includes all specimens of *Saxatilomys paulinae*) were preserved in 70% ethanol for later identification using museum mammal collections. Species identification follows Musser et al. (2005)​. All voucher specimens of rodents were cataloged and stored at zoological museums of Institute of Ecology and Biological Resources in Hanoi, Vietnam (DVZ-PNKB-01, DVZ-PNKB-03, DVZ-PNKB-04. DVZ-PNKB-07). Craniodental measurements of the voucher skulls were taken according to Musser and Newcomb (1983) with vernier calipers to the nearest 0.05 mm. Following measurements were taken from each specimen: occipitonasal length (ONL), zygomatic breadth (ZB), interorbital breadth (IB), length of rostrum (LR), breadth of rostrum (BR), breadth of braincase (BBC), height of braincase (HBC), breadth of zygomatic plate (BZP), length of diastema (LD), length of incisive foramina (LIF), breadth of incisive foramina (BIF), length of bony palate (LBP), breadth across bony palate at first molars (BBP), postpalatal length (PPL), breadth of mesopterygoid fossa (BMF), length of bulla (LB), crown length of maxillary molar row (CLM1-3) and breadth of first upper molar (BM1). The craniodental measurements are taken only from adult specimens. Species identification follows Lekagul and McNeeley (1988) and Francis (2008).

Study of habitat was carried out using transect and plot techniques (White and Edwards 2000). Plots of 10 x 10 m were used for inventory of all trees with height more than 3 m, plots of 4 x 4 m were used for inventory of bush trees of height from 0.5 m to 3 m and plots of 1 x 1 m were used for inventory of herbs and tree seedlings of less than 0.5 m high. Topographic features and surface ground substrates were also noted. Threats to rodent populations and habitat were evaluated based on interviews of local villagers and direct field observation of threat signs (traps, hunters, logging, forest clearing, human encroachment) in the survey area.

## Taxon treatments

### Saxatilomys paulinae

Musser, Smith, Robinson & Lunde, 2005

#### Materials

- **Type status:** Other material. **Occurrence:** recordedBy: Nghia Xuan Nguyen; individualCount: 1; sex: female; **Location:** country: Vietnam; stateProvince: Quang Binh; verbatimLocality: Thuong Hoa Commune, Minh Hoa District; verbatimElevation: 295 m; verbatimLatitude: 17°48’N; verbatimLongitude: 105°55’E; **Event:** eventDate: April 13, 2014; habitat: Karst forest; **Record Level:** collectionID: NXN-215; institutionCode: IEBR; collectionCode: DVZ-Rodentia; ownerInstitutionCode: IEBR
- **Type status:** Other material. **Occurrence:** recordedBy: Nghia Xuan Nguyen; individualCount: 1; sex: male (juv.); **Location:** country: Vietnam; stateProvince: Quang Binh; verbatimLocality: Thuong Hoa Commune, Minh Hoa District; verbatimElevation: 315 m; verbatimLatitude: 17°48’N; verbatimLongitude: 105°55’E; **Event:** eventDate: April 14, 2014; habitat: Karst forest; **Record Level:** collectionID: NXN-217; institutionCode: IEBR; collectionCode: DVZ-Rodentia; ownerInstitutionCode: IEBR
- **Type status:** Other material. **Occurrence:** recordedBy: Nghia Xuan Nguyen; individualCount: 1; sex: female; **Location:** country: Vietnam; stateProvince: Quang Binh; verbatimLocality: Thuong Hoa Commune, Minh Hoa District; verbatimElevation: 315 m; verbatimLatitude: 17°48’N; verbatimLongitude: 105°55’E; **Event:** eventDate: April 15, 2014; habitat: Karst forest; **Record Level:** collectionID: NXN-218; institutionCode: IEBR; collectionCode: DVZ-Rodentia; ownerInstitutionCode: IEBR
- **Type status:** Other material. **Occurrence:** recordedBy: Nghia Xuan Nguyen; individualCount: 1; sex: male; **Location:** country: Vietnam; stateProvince: Quang Binh; verbatimLocality: Thuong Hoa Commune, Minh Hoa District; verbatimElevation: 298 m; verbatimLatitude: 17°48’N; verbatimLongitude: 105°55’E; **Event:** eventDate: April 17, 2014; habitat: Karst forest; **Record Level:** collectionID: NXN-221; institutionCode: IEBR; collectionCode: DVZ-Rodentia; ownerInstitutionCode: IEBR

#### Description

**Figure 1.**
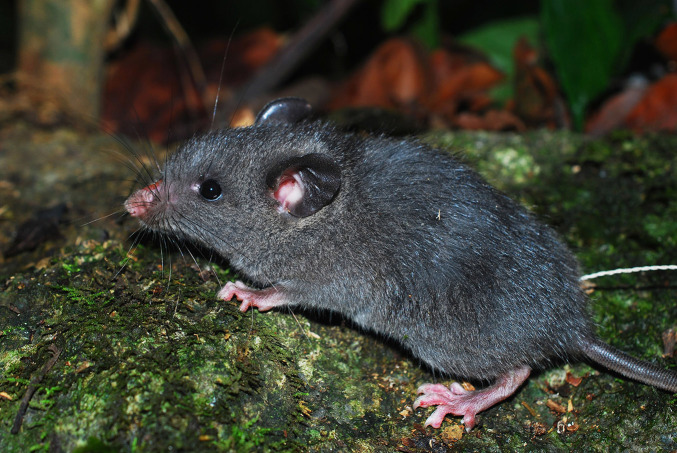
Living specimen of *Saxatilomys paulinae* from Quang Binh province (the nose is wounded by trapping).

**Figure 2.**
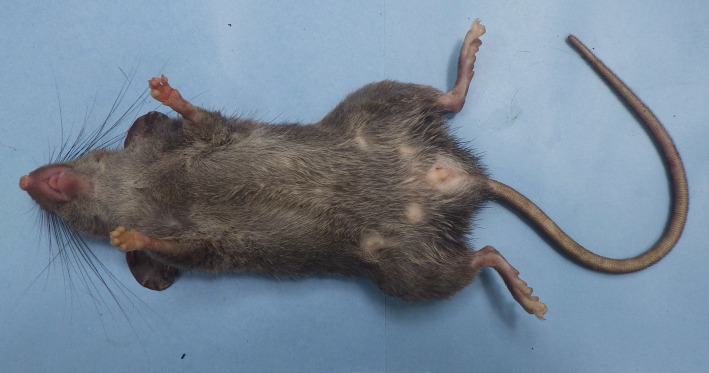
Ventral view of *Saxatilomys paulinae* from Quang Binh province.

**Figure 3.**
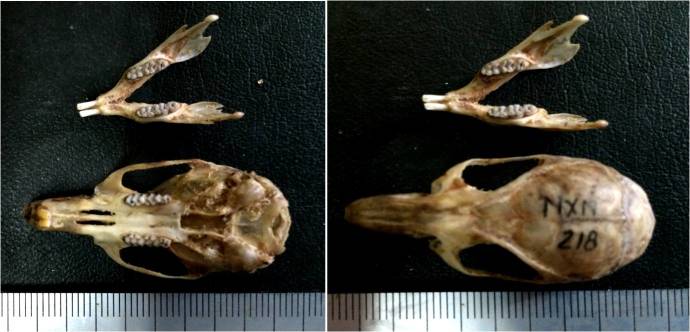
Skull of adult *S. paulinae*.

Four whole specimens of *Saxatilomys paulinae* (two adult females, one adult male and one juvenile male) were collected in April 2014. The external and craniodental measurements of these specimens are shown in Table 1. The external and craniodental characteristics of all specimens fit well the diagnosis of *Saxatilomys paulinae* described in Musser et al. (2005). The rats have medium size (HB: 160.3 ± 2.03 mm, T: 192.3 ± 6.69 mm) with the following key specific characteristics: semispinous dark gray upperparts, dark frosted gray underparts, no sharp demarcation between dorsal and ventral fur and no white patches on the body (Fig. 1 and Fig. 2). Each palmar surface is covered with five huge and swollen pads while each plantar surface is covered with 6 very large and extremely bulbous pads (Fig. 3​). Tail is slim, round in cross-section, gray in dorsal and lateral surfaces, whitish (un-pigmented) in ventral surface. The tail length is significantly longer than head-body length (adult: 113-129%, juvenile: 149%). Skull is elongated with narrow rostrum and wide braincase.

#### Distribution

**Figure 4.**
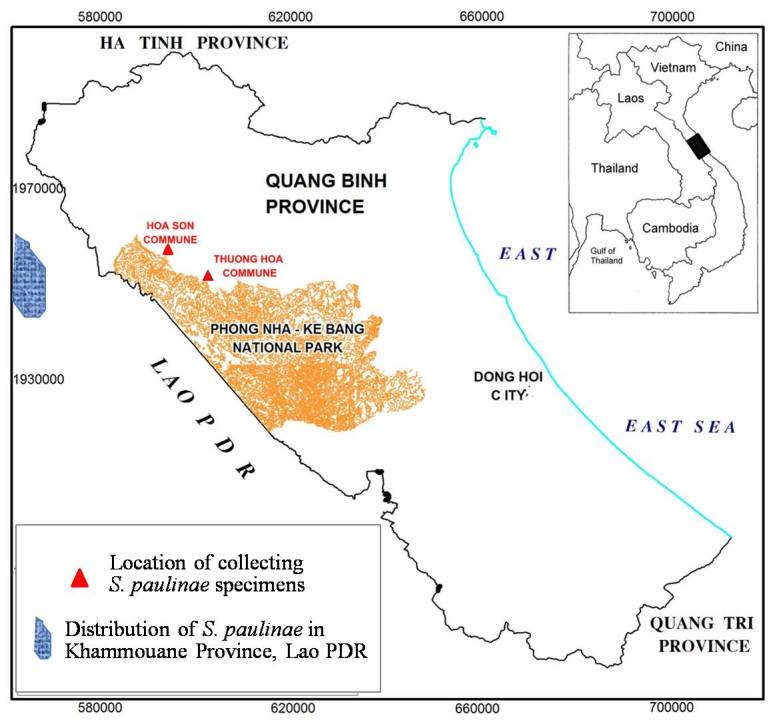
Location of survey areas (the red commune names) and locality where specimens of *Saxatilomys paulinae* were collected.

The specimens of *Saxatilomys paulinae* were captured only in Thung Uc locality (17°48' N, 105°55' E) of Thuong Hoa Commune (Fig. 4​). Local villagers reported they have captured this species in several localities near 3 villages of Thuong Hoa Commune (Ban On, Yen Hop and Mo-O villages). However, this information have not yet been verified by our trapping survey.

#### Ecology

**Figure 5.**
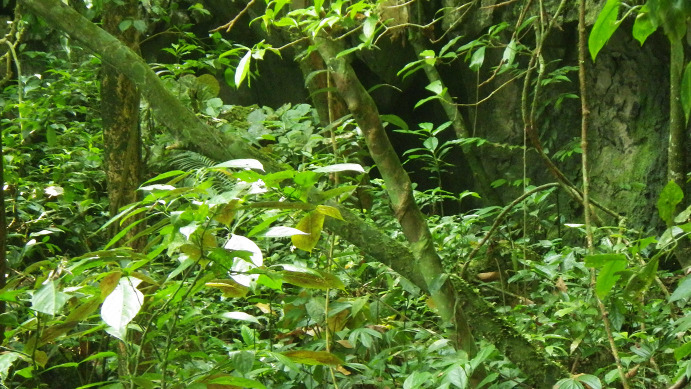
Microhabitat of *S. paulinae* in Thuong Hoa Commune.

All specimens of *Saxatilomys paulinae* were collected at the base of a Thung Uc karst tower (Thuong Hoa Commune), at elevation of 295 - 315 m, under limestone humid evergreen forest (Fig. 5). This habitat type covers larger area in Thuong Hoa Commune where the species occurence was reported by local residents.

The habitat is characterized by complicated terrain comprising low karst towers (around 400 m) with steep slopes covered under limestone humid evergreen forest. The slopes have many large limestone boulders and crevices. The forest on the slopes has been affected by selected timber logging in the past; however, a 3-4 layer forest structure remains, with the following characters:

The canopy tree layer consists of trees 20-30 m high with stem diameter 0.5-0.8 m. The most common trees species are: *Pometia pinnata* (Sapindaceae), *Dracontomelon duperreanum* (Anacardiaceae), *Toona surenii* (Meliaceae), *Paviesia anamensis* (Sapindaceae), *Pterospermum grewiaefolium* (Sterculiaceae), *Mahuca* sp., *Hopea* sp., *Streblus asper* (Moraceae), *Litsea* sp. (Lauraceae), *Sumbaviopsis macrophylla* (Euphorbiaceae), *Actinodaphne* sp. (Lauraceae), *Pometia chinensis* (Sapindaceae), *Choerospondias axillaris* (Anacardiaceae), *Alangium ridleyi* (Alangiaceae), *Knema* sp. (Myristicaceae), etc.

The middle tree layer consists of trees 10-15 m high with stem diameter 0.3-0.5 m. The most common species are *Knema corticosa* (Myristicaceae), *Streblus tonkinensis*, *Streblus asper* (Moraceae), *Xylopia vielana* (Annonaceae), *Diospyros* sp. (Ebenaceae), *Caryota mitis* (Arecaceae), *Arenga pinnata* (Arecaceae), *Camelia* sp. (Theaceae), *Actinodaphne* sp. (Lauraceae), *Pterospermum* sp. (Sterculiaceae), *Litsea* sp. (Lauraceae), *Ormosia laoensis* (Fabaceae), *Nephelium lappaceum* (Sapindaceae), *Sumbaviopsis macrophylla* (Euphorbiaceae), *Paranephelium spirei* (Sapindaceae), *Alangium ridleyi* (Alangiaceae), *Baccaurea* sp. (Euphorbiaceae), *Aglaia* sp. (Meliaceae), etc.

The scrub layer consists of trees 3-7 m high, mostly with twisted stems, many branches, and several stems rising from one base. The most common species are from the families Euphorbiaceae, Theaceae, Myrtaceae and Verbenaceae. Some dominant species are *Antidesma* sp. (Euphorbiaceae), *Trevesia panmalta* (Araliaceae), *Litsea valiabilis* (Lauraceae), *Arenga pinnata* (Arecaceae), *Excoecaria cochinchinensis* (Euphorbiaceae), as well as seedlings of trees from higher layers.

The herb and fern layer is about 0.5-3 m high, with trees of 0.2-3 m high from family Araceae, the genera *Calamus* and *Caryota* (family Arecaceae), and many herb species from families Urticaceae, Melastomataceae, Balsaminaceae, Poaceae, Begoniaceae, Podipoliaceae, Convallariaceae, Zingiberaceae, Urticaceae and Acanthaceae. Some of the most common species are *Homalomena occulta* (Araceae), *Aglaonema simplex* (Araceae), *Aglaonema siamensis* (Araceae), *Tacca chantrieri* (Taccaceae), *Aspidistra typica* (Convallariaceae), *Piper* sp. (Piperaceae), *Corymborkis veratrifolia* (Orchidaceae), etc.

Apart from *Saxatilomys paulinae*, several other ground-living rodent species are found in this habitat including *Bandicota indica*, *Berylmys bowersi*, *Leopoldamys sabanus*, *Leopoldamys edwardsi*, *Maxomys moi*, *Maxomys surifer*, *Niviventer fulvescens*, *Niviventer langbianis*, *Niviventer tenaster*, *Rattus tanezumi*, *Rattus andamanensis*, and *Laonastes aenigmamus* (Nguyen et al. 2013).

#### Conservation

Main threats to the population of *Saxatilomys paulinae* in Quang Binh province (Vietnam) is wildlife hunting and habitat disturbance. The distribution area of the *Saxatilomys paulinae* is situated close to the villages of ethnic minorities (Ruc, Sach, and Chut). These minority groups are very poor and their livelihood much depends on wildlife and forest products. Wildlife hunting is a long tradition of the local people, and a practice that remains extensive currently. Most men 15 to 60 years in age in these villages are engaged in wildlife hunting. Their hunting season lasts about eight months per year (from July to February). The most widely used mean for trapping rodents is metal spring snares. Each hunter usually keeps 30-100 active snares in forests; some hunters keep up to 300-500 active snares. Unfortunatlely, we were not able to estimate how many individuals of *Saxatilomys paulinae* they capture each year.

## Discussion

Simple comparison of external and craniodental measurement of specimens from Vietnam with those of specimens from Lao indicates that specimens from Vietnam are generally larger than specimens from Lao (Table 1​). However, due to very small number of specimens examined, this needs to be verified by more studies in future.

In Lao, *Saxatilomys paulinae* was reported to inhabit steep rocky slopes with large limestone boulders covered in heavily degraded deciduous forest mixed with scrub and bamboo at the base of the surrounding massive karst (Musser et al. 2005). Vietnam's population of *Saxatilomys paulinae* was found in rocky slopes with large limestone boulders, under the limestone humid evergreen forest which is different from the deciduous forest type in Lao. This indicates *Saxatilomys paulinae* can tolerate different limestone forest habitats existing in the Central Indochina Limestone landscape.

Before this study, *Saxatilomys paulinae* was recorded only in the Phoun Hin Poun NBCA in Khammuoane province of Lao (Musser et al. 2005). Our records of *Saxatilomys paulinae* in Quang Binh province expand global distribution range of this species into Vietnam's part of Central Indochina Limestone for about 100 km east-ward. However, the range of the species distribution remains restricted by only five known localities (four localities in Lao and one locality in Vietnam), while trapping and habitat disturbance remain as current threats to the species survival. More surveys need to be conducted to assess the species distributional range and the status of its populations for undertaking relevant conservation measures.

It is interesting that both *Saxatilomys paulinae* and *Laonastes aenigmamus* share the same limestone forest habitat in Central Indochina Limestone. The *Laonastes aenigmamus* is the only surviving member of the otherwise extinct rodent family Diatomyidae, that was formerly believed to have been extinct for more than 11 million years (Dawson et al. 2006). Both species are currently known only from few localities in Central Indochina Limestone. This again indicates high importance of the Central Indochina Limestone for the global biodiversity conservation.

## Supplementary Material

XML Treatment for Saxatilomys paulinae

## Figures and Tables

**Table 1.** External and crania-dental measurements of *Saxatilomys paulinae* specimens from Vietnam and those of specimens from Laos. Note: * statistics of 3 adult specimens (NXN-215, NXN-218 and NXN-221) collected in Phong Nha - Ke Bang NP; ** data from specimens collected in Phoun Hin Poun NBCA, Lao PDR (in Musser et al. 2005). Mean, plus or minus one standard deviation, observed range in parentheses and number of specimens in each sample are listed for each measurement. All measurements are in milimetre except body weight (W) in grams.

**Measu** **rement**	**Specimens from Vietnam**				**Statistics of specimens** from Vietnam*	**Statistics of specimens** from Lao**
	**NXN-217**	**NXN-221**	**NXN-218**	**NXN-215**		
**Sex**	**♂ (juv.)**	**♂ (adult)**	**♀ (adult)**	**♀ (adult)**	**♂ & ♀**	**♂ & ♀**
HB	116	157	160	164	160.33 ± 2.03 (157-164) 3	(144-150) 2
T	164	203	180	194	192.33 ± 6.69 (180-203) 3	(167-168) 2
T/HB (%)	141	129	113	118	(113-129) 3	(112-116) 2
HF	28.42	29.68	29.25	30	29.64 ± 0.22 (29.25-30.0)	32-32
E	23.52	24.96	25.18	25.42	25.19 ± 0.13 (24.96-25.42) 3	(24-25) 2
W	50	130	110	110	116.67 ± 6.67 (110-130) 3	
ONL	36.54	43.69	42.71	42.81	43.07 ± 0.31 (42.71-43.69) 3	36.0 ± 1.37 (33.3-40.5) 48
ZB	16.8	19.3	19.03	18.79	19.04 ± 0.15 (18.79-19.3) 3	16.9 ± 0.62 (15.7-18.9) 48
IB	6.0	6.18	6.43	6.39	6.33 ± 0.08 (6.18-6.43) 3	5.8 ± 0.29 (5.1-6.5) 48
LR	11.34	14.2	13.57	13.97	13.91 ± 0.18 (13.57-14.2) 3	11.0 ± 0.65 (9.7-12.7) 48
BR	5.26	6.74	6.63	6.62	6.66 ± 0.04 (6.62-6.74) 3	5.9 ± 0.31 (5.3-6.7) 48
BBC	15.87	16.27	16.53	16.02	16.27 ± 0.15 (16.02-16.53) 3	15.0 ± 0.36 (14.3-16.2) 48
HBC	15.87	16.27	16.53	16.02	16.27 ± 0.15 (16.02-16.53) 3	9.9 ± 0.36 (9.0-10.9) 48
BZP	3.5	4.77	4.41	4.52	4.57 ± 0.11 (4.41-4.77) 3	3.4 ± 0.26 (2.9-4.0) 48
LD	8.19	11.69	11	10.63	11.11 ± 0.31 (10.63-11.69) 3	9.1 ± 0.54 (7.9-10.4) 48
LIF	6.36	7.61	6.42	7.1	7.04 ± 0.34 (6.42-7.61) 3	6.6 ± 0.45 (5.6-7.5) 48
BIF	2.24	2.49	2.43	2.33	2.42 ± 0.05 (2.33-2.49) 3	2.6 ± 0.19 (2.2-3.1) 48
LBP	8	9.47	9.84	9.54	9.62 ± 0.11 (9.47-9.84)	6.1 ± 0.36 (5.3-6.9) 48
BBP	3.06	3.69	3.89	3.45	3.68 ± 0.13 (3.45-3.89) 3	3.3 ± 0.19 (3.0-3.9) 48
PPL	10.74	13.72	12.61	12.81	13.05 ± 0.34 (12.61-13.72) 3	12.3 ± 0.68 (10.9-14.5) 48
BMF	2.5	2.83	2.46	2.43	2.57 ± 0.13 (2.43-2.83) 3	2.7 ± 0.24 (2.3-3.4) 48
LB	5.38	5.79	5.32	5.57	5.56 ± 0.14 (5.32-5.79) 3	5.4 ± 0.18 (5.5-5.8) 48
CLM1-3	7.7	7.81	7.57	7.86	7.75 ± 0.09 (7.57-7.86) 3	6.2 ± 0.17 (5.8-6.5) 48
BM1	2.08	2.03	1.95	1.97	1.98 ± 0.02 (1.95-2.03) 3	1.8 ± 0.08 (1.7-2.0) 48
